# Can preoperative visual analogue scale chart patterns predict surgical outcomes in older adults with lumbar spinal stenosis? A two-center retrospective study

**DOI:** 10.20407/fmj.2025-015

**Published:** 2025-11-05

**Authors:** Kohei Shibata, Soya Kawabata, Takehiro Michikawa, Yuki Akaike, Yukio Nakajima, Sota Nagai, Kurenai Hachiya, Takaya Imai, Hiroki Takeda, Atsushi Yoshioka, Shinjiro Kaneko, Yudo Hachiya, Nobuyuki Fujita

**Affiliations:** 1 Department of Orthopaedic Surgery, School of Medicine, Fujita Health University, Toyoake, Aichi, Japan; 2 Department of Orthopaedic Surgery, Hachiya Orthopaedic Hospital, Nagoya, Aichi, Japan; 3 Department of Environmental and Occupational Health, School of Medicine, Toho University, Ota, Tokyo, Japan; 4 Department of Orthopaedic Surgery, Keio University, School of Medicine, Shinjuku, Tokyo, Japan; 5 Department of Spine and Spinal Cord Surgery, School of Medicine, Fujita Health University, Toyoake, Aichi, Japan

**Keywords:** Lumbar spinal stenosis, Visual analogue scale, Japanese Orthopaedic Association Back Pain Evaluation Questionnaire, Zurich Claudication Questionnaire, Patient satisfaction

## Abstract

**Objectives:**

Patients with lumbar spinal stenosis (LSS) exhibit significantly different scoring patterns on the visual analogue scale (VAS) chart for low back pain (LP), buttock and lower limb pain (PL), and buttock and lower limb numbness (NL). This study investigated the usefulness of these preoperative scoring patterns on the VAS chart in predicting surgical outcomes in older adults undergoing LSS surgery.

**Methods:**

Time-course data from patients aged ≥65 years who underwent LSS surgery at two institutions were retrospectively assessed. All participants completed the Zurich Claudication Questionnaire and the Japanese Orthopaedic Association Back Pain Evaluation Questionnaire, which included the VAS chart, before surgery and at 6 months and 1 year postoperatively.

**Results:**

In total, 334 participants were evaluated. Patients with equal preoperative scores across all three scales showed the highest average postoperative reduction in the three VAS scores. By contrast, those with the highest preoperative VAS scores for LP or NL had the lowest reductions. Based on the multivariable analysis, the highest preoperative VAS scores for LP (relative risk: 2.1) and NL (relative risk: 2.1) were significantly associated with poor surgical improvement in older adults with LSS.

**Conclusions:**

This study demonstrated the potential clinical utility of the preoperative VAS chart in predicting surgical improvement in older patients with LSS. Patients with equal preoperative VAS scores for LP, PL, and NL were more likely to have favorable surgical outcomes, while those with the highest preoperative scores for LP or NL were at higher risk for poor outcomes.

## Introduction

Lumbar spinal stenosis (LSS) is a prevalent degenerative musculoskeletal condition in older adults, with its incidence rising globally due to population aging.^1,2^ Common symptoms of LSS include low back and buttock pain, as well as leg pain and numbness, which often lead to considerable limitations in daily activities.^3,4^ Consequently, most older adults with LSS are regarded as being in a condition that may reduce their ability to maintain a healthy life expectancy.^5^

In clinical practice, the visual analogue scale (VAS) is a common tool for assessing chronic pain. It evaluates specific patient sensations on a scale from 0 to 10 and can readily detect subtle differences because of its continuous scale. In 1974, Huskisson^6^ reported that the VAS could serve as a standard tool for pain assessment. Since then, it has been widely used to evaluate pain across various patient populations.^7^ Currently, patient-reported outcomes assessed using tools such as the Oswestry Disability Index, Roland-Morris Disability Questionnaire, and Zurich Claudication Questionnaire (ZCQ) are employed to examine LSS symptoms.^3,4^ Additionally, the Japanese Orthopaedic Association Back Pain Evaluation Questionnaire (JOABPEQ)—a tool for evaluating patient-reported outcomes—includes a VAS chart comprising three scales to assess low back pain (LP), buttock and lower limb pain (PL), and buttock and lower limb numbness (NL).^8^ When older patients with LSS are asked to score their symptoms on this VAS chart, the resulting scoring patterns vary widely. For instance, some patients report the highest scores for NL, while others show the highest for LP; some may even score all three symptoms equally. A similar variation in symptom patterns is observed among older patients with LSS who undergo surgery. LP is known to be difficult to treat, and NL often persists after LSS surgery. Although several studies have evaluated longitudinal changes in the three VAS scales in LSS,^9–13^ it remains unclear whether preoperative scoring patterns on the VAS chart can predict surgical outcomes in older patients with LSS. This study aimed to longitudinally validate the detailed characteristics of the three VAS scales in older patients with LSS who underwent surgery, with particular focus on investigating the usefulness of preoperative scoring patterns on the VAS chart in predicting surgical outcomes.

## Materials and Methods

### Participants

Time-course data from patients aged ≥65 years who underwent LSS surgery at two institutions between April 2020 and July 2023 were retrospectively reviewed. Surgical indications and treatment procedures were determined by board-certified spine surgeons, based on patients’ symptoms and imaging results—including magnetic resonance imaging, computed tomography scans, and myelography—in accordance with the guidelines.^3,4^ Patients with an upper instrumented thoracic spine level or a lower instrumented pelvis level were excluded from the analysis.

### Ethics approval

The ethics committees of each participating institution approved this study. All eligible patients were included, except those who chose to opt out. The study was conducted in accordance with the guidelines of the Declaration of Helsinki.

### Data collection

All participants completed the ZCQ and JOABPEQ, which included the VAS chart, before surgery and at 6 months and 1 year postoperatively. The ZCQ consists of three subscales: symptom severity, physical function, and patient satisfaction.^14^ Data collected included age, sex, body mass index (BMI), medical history (e.g., diabetes mellitus, hypertension, dyslipidemia, cardiovascular disease, stroke, and cancer), presence of spondylolisthesis, degenerative lumbar scoliosis, failed back surgery syndrome (FBSS), American Society of Anesthesiologists (ASA) physical status, radiographic parameters, and perioperative variables such as type of surgical procedure, number of operated levels, surgical duration, and intraoperative blood loss volume. Radiographic parameters included sagittal vertical axis, thoracic kyphosis, pelvic tilt, pelvic incidence, and lumbar lordosis, as measured on standing full-length plain radiographs.

### VAS

**Figure 1:**
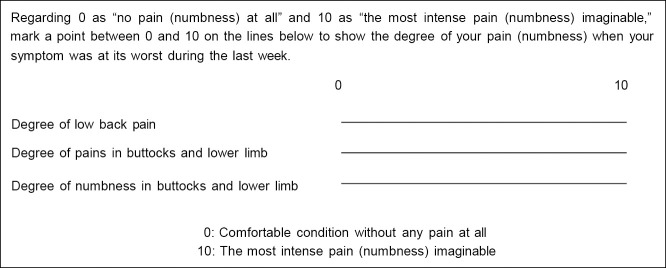
Visual analogue scale (VAS) chart included in the Japanese Orthopaedic Association Back Pain Evaluation Questionnaire (JOABPEQ).

**Figure 2:**
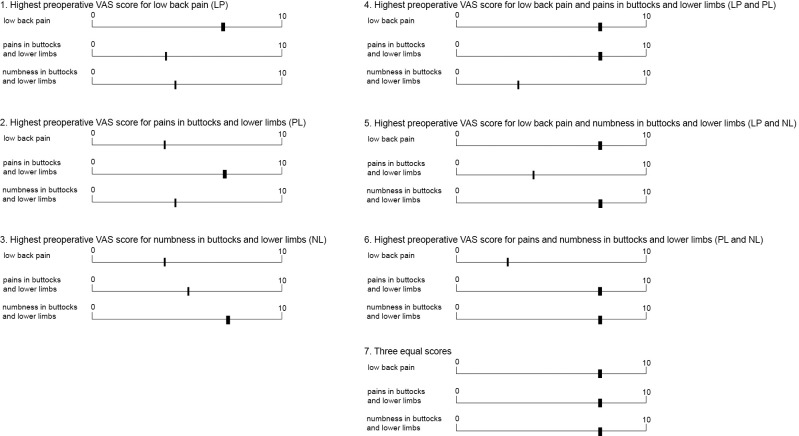
Preoperative scoring patterns on the visual analogue scale (VAS) chart.

The VAS scores for LP, PL, and NL were individually assessed using the VAS chart included in the JOABPEQ (Figure 1). If numerical values were indicated on the VAS chart, those values were used as the VAS score. If no numerical values were provided, the mark on the line was measured from the leftmost 0-mark using a ruler, and the measured length was converted to a VAS score by scaling it to a range of 0 to 10. In such cases, the scores were recorded to the first decimal place. Accordingly, all VAS scores in this study were presented to one decimal point, and any difference of ≥0.1 was treated as a distinct VAS value. Based on previous literature,^15^ poor surgical improvement was defined as an average reduction of <2.0 across the three VAS scores at 1 year postoperatively. Figure 2 shows the scoring patterns of the three preoperative VAS scores. According to the VAS scores for LP, PL, and NL, patients were classified into the following groups: group 1 included patients with the highest preoperative VAS score for LP; group 2, those with the highest score for PL; group 3, those with the highest score for NL; group 4, those whose scores for LP and PL were identical and higher than that for NL; group 5, those whose scores for LP and NL were identical and higher than that for PL; group 6, those whose scores for PL and NL were identical and higher than that for LP; and group 7, those with identical scores across all three scales.

### Statistical analyses

Data among the groups were compared using the Wilcoxon signed-rank test or the Kruskal–Wallis test with Bonferroni correction, as appropriate. To assess the correlation between two parameters, Pearson’s correlation coefficient (r) was calculated. Analyses were conducted using the Statistical Package for the Social Sciences software version 21.0 (IBM Corp., Armonk, NY, USA). We explored factors associated with poor surgical improvement, including age, sex, BMI, scoring patterns of the three preoperative VAS scores, diabetes mellitus, ASA physical status, spondylolisthesis, degenerative lumbar scoliosis, FBSS, type of surgical procedure, number of operated levels, surgical duration, intraoperative blood loss volume, and radiographic parameters such as sagittal vertical axis, thoracic kyphosis, and pelvic incidence–lumbar lordosis. A Poisson regression model was then constructed, adjusted for age, sex, and factors that were statistically significant in the univariable model, to estimate relative risks (RRs) and 95% confidence intervals (CIs) for poor surgical improvement. Poisson regression analysis was performed using STATA16 software (StataCorp, College Station, TX, USA). All p values of <0.05 were considered statistically significant.

## Results

In total, 334 participants were assessed. Table 1 shows the baseline characteristics of the participants. Postoperative ZCQ and JOABPEQ scores (at 6 months and 1 year) were significantly improved compared with the preoperative data (Table 2). Next, changes in VAS scores for LP, PL, and NL before and after surgery were examined (Table 3). All three VAS scores significantly decreased at both 6 months and 1 year postoperatively. A cross-sectional analysis revealed that the preoperative VAS score for LP was significantly lower than that for PL. At 1 year postoperatively, the VAS score for NL was marginally higher than those for LP and PL. However, there were no significant differences among the postoperative VAS scores for LP, PL, and NL. The decrease in VAS scores for LP and NL at 1 year postoperatively was significantly smaller than that for PL. Next, the correlation between the reduction in each VAS score at 1 year postoperatively and the ZCQ satisfaction scores was examined (Table 4). The VAS score for PL showed the strongest correlation with satisfaction (r=–0.28). However, the correlations between each of the three VAS scores and satisfaction were weak. The correlation between the average reduction in the three VAS scores and the ZCQ satisfaction score was –0.29 (Table 4).

**Figure 3:**
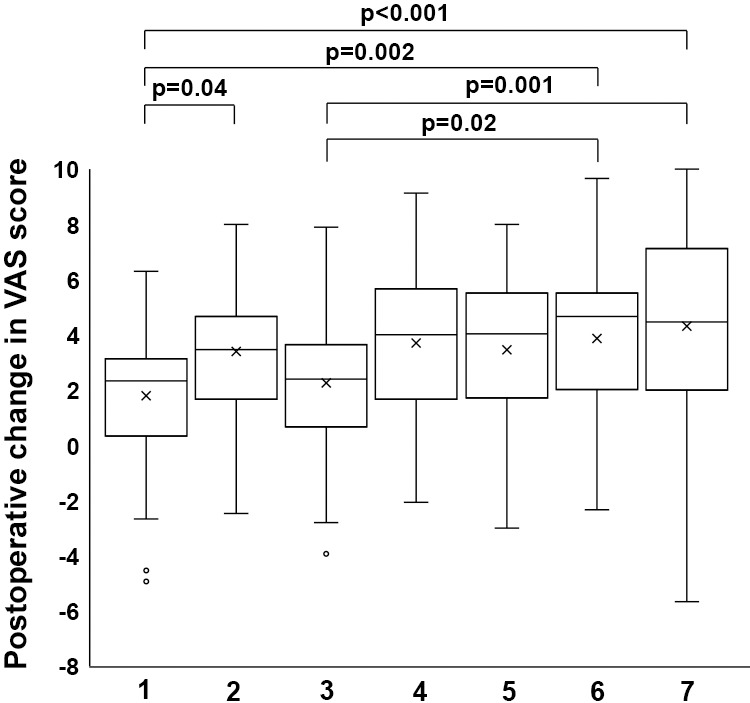
Box plot showing the average reduction in the three visual analogue scale (VAS) scores at 1 year postoperatively across the seven groups. 1. Highest preoperative VAS score for low back pain (LP) (n=59), 2. Highest preoperative VAS score for pains in buttocks and lower limbs (PL) (n=63), 3. Highest preoperative VAS score for numbness in buttocks and lower limbs (NL) (n=70), 4. Highest preoperative VAS score for low back pain and pains in buttocks and lower limbs (LP and PL) (n=37), 5. Highest preoperative VAS score for low back pain and numbness in buttocks and lower limbs (LP and NL) (n=17), 6. Highest preoperative VAS score for pains and numbness in buttocks and lower limbs (PL and NL) (n=46), 7. Three equal scores (n=52)

Next, the average reduction in the three VAS scores at 1 year postoperatively was compared across the seven groups based on the patterns shown on the VAS chart (Figure 3). The preoperative and 1-year postoperative VAS scores, along with their changes in each group, are summarized in Table 5. The highest average reduction in the three VAS scores was observed in group 7, where all three preoperative scores were initially the same. By contrast, the lowest average reductions were seen in group 1, which had the highest preoperative score for LP, and group 3, which had the highest score for NL (Figure 3). Statistically, the value for group 1 was significantly lower than that for group 2 (p=0.04), group 6 (p=0.002), and group 7 (p<0.001) (Figure 3). The value for group 3 was significantly lower than that for group 6 (p=0.02) and group 7 (p=0.001) (Figure 3).

The factors associated with poor surgical improvement were examined using a Poisson regression model (Table 6). In this analysis, the incidence of poor surgical improvement was similar among groups 4, 5, and 6, and the sample size for each was small, so these three groups were combined. Based on the multivariable analysis, a BMI of ≥25 kg/m^2^ (RR: 1.4 compared with a BMI of <25 kg/m^2^, 95% CI: 1.1–1.9), group 1 (RR: 2.1 compared with group 7, 95% CI: 1.1–3.8), and group 3 (RR: 2.1 compared with group 7, 95% CI: 1.2–3.7) were significantly associated with poor surgical improvement.

Finally, a separate analysis limited to patients who underwent decompression alone was conducted, as shown in Supplementary Table 1. Although no statistically significant associated factors were identified, the proportion of patients classified into group 1 and group 3 tended to be higher in the poor surgical improvement group.

## Discussion

This study simultaneously evaluated LP, PL, and NL in older patients with LSS using a VAS chart to validate the detailed characteristics of their longitudinal changes and to identify preoperative scoring patterns on the VAS that were associated with poor postoperative outcomes. Regarding the longitudinal changes in the three VAS scores, the surgical reduction in PL was significantly greater than that in LP and NL, which is consistent with previous research findings.^9,16,17^ Additionally, among the three VAS scales, patient satisfaction with surgery was most strongly correlated with the reduction in the VAS score for PL. Nevertheless, this correlation was not particularly strong. Moreover, patient satisfaction was only weakly correlated with the average reduction in the three VAS scores. This suggests that patient satisfaction does not solely depend on changes in VAS scores. Previous studies have reported that satisfaction is also influenced by improvements in walking ability and preoperative psychological conditions.^18–20^ Therefore, patient satisfaction is likely determined by a complex set of factors.

In this study, there were seven distinct scoring patterns on the VAS chart. The most frequent pattern was the highest preoperative VAS score for NL, followed by the highest preoperative score for PL and then LP. Among these three patterns, patients with the highest preoperative VAS score for PL showed the most favorable improvement in average postoperative VAS scores. By contrast, those with the highest preoperative score for LP or NL exhibited the least improvement. These results are partially consistent with the notion that LP and NL are less likely to improve and more likely to persist after LSS surgery.^9,16,17^ Conversely, the reason why patients with three equal preoperative scores experienced the greatest improvement remains unclear. Based on the multivariable analysis, using patients with three equal scores as the reference, having the highest preoperative VAS score for NL or LP was significantly associated with poor postoperative outcomes. These findings suggest that the VAS chart may be a useful tool for clinicians to communicate outcome tendencies to older patients with LSS during the preoperative informed consent process, offering clinical value. Additionally, our analysis showed that a higher BMI was associated with poor postoperative outcomes, consistent with previous reports.^21,22^ Although previously reported predictors of poor surgical outcomes—such as ASA physical status,^23^ diabetes mellitus,^24^ age,^25^ FBSS,^23^ degenerative lumbar scoliosis,^10^ and sagittal imbalance^26^—were included as explanatory variables in the multivariate analysis, no significant associations were found. Discrepancies between our findings and earlier studies may reflect differences in how poor surgical outcomes are defined or variations in patient populations. Further studies with larger sample sizes and multi-faceted analyses are needed to validate these results. Taken together, the preoperative scoring pattern on the VAS chart can serve as a partial predictor of postoperative outcomes in older patients with LSS. As the use of electronic media for entering patient-reported outcomes increases, incorporating larger datasets along with additional factors such as age, sex, and BMI—and applying machine learning techniques to predict postoperative VAS scores—may hold clinical value.

This study had several limitations. First, a floor effect was present in the reduction of VAS scores. Therefore, if preoperative VAS scores were already low, the potential for postoperative reduction may have been limited. Second, the follow-up period was only 1 year. However, extending the observation period in older patients increases the likelihood that other conditions unrelated to LSS may influence outcomes, making it difficult to exclusively assess the effects of LSS surgery. Third, the study did not exclude cases of spondylolisthesis or degenerative lumbar scoliosis, nor did it focus solely on patients undergoing decompression surgery alone, resulting in a heterogeneous study population. Fourth, medication use, physical therapy, and other non-surgical interventions were not considered as potential confounding factors influencing postoperative outcomes. Despite these limitations, the study clearly demonstrated the potential clinical utility of the VAS chart in predicting surgical improvement for older patients with LSS.

In conclusion, our study examined the detailed longitudinal characteristics of VAS scores for LP, PL, and NL in older patients with LSS who underwent surgery. Patients with three equal scores for the symptoms on the preoperative VAS chart were more likely to show favorable improvement in VAS scores after surgery. By contrast, those with the highest scores for LP or NL were at greater risk for poor surgical outcomes. These findings may assist in managing the complex symptoms of older patients with lumbar spinal stenosis.

## Figures and Tables

**Table 1:** Baseline characteristics

Patients		n=334
Sex		Male: 173, Female: 161
Age (years)		76.1±5.9
BMI (kg/m^2^)		23.7±3.5
Medical history	Diabetes mellitus	91 (27.2%)
	Hypertension	230 (68.9%)
	Dyslipidemia	152 (45.5%)
	Cardiovascular disease	96 (28.7%)
	Stroke	38 (11.4%)
	Cancer	53 (15.9%)
Spondylolisthesis		129 (38.6%)
Degenerative lumbar scoliosis		64 (19.2%)
FBSS		31 (9.3%)
Radiographic parameters	SVA (mm)	56.6±46.0
	TK (°)	31.5±11.6
	PT (°)	23.5±8.7
	PI-LL (°)	15.0±13.8
Surgical procedure	Without fusion	181 (54.2%)
	With fusion	153 (45.8%)
Operated levels	1	107 (32.0%)
	2	113 (33.8%)
	≥3	114 (34.1%)
Surgical time (min)		149.0±99.8
Surgical blood loss (mL)		175.1±181.1

Data are presented as n (%) or mean±standard deviation. BMI, body mass index; FBSS, failed back surgery syndrome; SVA, sagittal vertical axis; TK, thoracic kyphosis; PI, pelvic incidence; PT, pelvic tilt; LL, lumbar lordosis

**Table 2:** Patient-reported outcomes at baseline and follow-up (n=334)

		Median (25th–75th percentile)			p value^a^	
		Preop	6 mos Postop	1 year Postop	Preop vs. 6 mos Postop	Preop vs. 1 year Postop
ZCQ	Symptom severity	3.4 (2.9–3.9)	2.4 (1.9–3.0)	2.4 (1.9–2.3)	<0.01	<0.01
	Physical function	2.6 (2.0–3.0)	1.8 (1.2–2.2)	1.6 (1.2–2.2)	<0.01	<0.01
JOABPEQ	Pain disorder	43 (14–71)	100 (43–100)	100 (43–100)	<0.01	<0.01
	Lumbar function	50 (25–75)	75 (42–98)	75 (50–92)	<0.01	<0.01
	Walking ability	21 (7–43)	64 (29–93)	64 (36–93)	<0.01	<0.01
	Social life	38 (24–51)	51 (43–73)	57 (46–78)	<0.01	<0.01
	Psychological disorder	42 (32–53)	53 (45–67)	54 (45–69)	<0.01	<0.01

Preop, preoperatively; Postop, postoperatively ZCQ, Zurich Claudication Questionnaire; JOABPEQ, JOA Back Pain Evaluation Questionnaire ^a^ Wilcoxon signed-rank test

**Table 3:** Preoperative and postoperative visual analogue scale scores (n=334)

	LP	PL	NL	p value^b^
Preop	5.6±2.8^c^	6.3±2.8	6.0±3.0	0.003
6 mos Postop	2.7±2.4	2.6±2.8	3.0±3.0	0.305
1 year Postop	2.7±2.6	2.6±2.7	3.2±3.1	0.054
p value^a^ (Preop vs. 6 mos Postop)	<0.001	<0.001	<0.001	
p value^a^ (Preop vs. 1 year Postop)	<0.001	<0.001	<0.001	
Reduction amount at 1 year Postop	2.9±3.2^c^	3.7±3.5	2.8±3.6^c^	<0.001

Preop, preoperatively; Postop, postoperatively; LP, low back pain; PL, buttock and lower limb pain; NL, buttock and lower limb numbness ^a^ Wilcoxon signed-rank test ^b^ Kruskal–Wallis test ^c^ p<0.05 (Bonferroni correction; reference=PL)

**Table 4:** Correlation between patient satisfaction and reduction in VAS scores (n=334)

	Amount of reduction in low back pain	Amount of reduction in buttock and lower limb pain	Amount of reduction in buttock and lower limb numbness	Average amount of reduction in the three VAS scores
Correlation coefficient	–0.20	–0.28	–0.21	–0.29
p value	<0.001	<0.001	<0.001	<0.001

VAS, visual analogue scale

**Table 5:** Poisson regression model of poor surgical improvement

			Patients (n)	Incidents (n)	Incidence (%)	Univariable model				Multivariable model^a^			
						Relative risk	95% Confidence interval		p-value	Relative risk	95% Confidence interval		p-value
Age (years)		65–69	85	15	25.9	Reference				Reference			
		70–79	183	62	33.9	1.3	0.8	2.1	0.27	1.2	0.8	2.0	0.40
		≥80	93	35	37.6	1.5	0.9	2.4	0.15	1.3	0.8	2.1	0.32
Sex		Male	173	53	30.6	Reference				Reference			
		Female	161	59	36.7	1.2	0.9	1.6	0.25	1.2	0.9	1.6	0.33
BMI (kg/m^2^)		<25	228	67	29.4	Reference				Reference			
		≥25	106	45	42.5	1.4	1.1	1.9	0.02	1.4	1.0	1.8	0.04
Distribution patterns of the three preoperative visual analogue scale scores		1	59	27	45.8	2.2	1.2	3.9	0.01	2.1	1.1	3.8	0.02
		2	63	19	30.2	1.4	0.7	2.7	0.28	1.4	0.7	2.7	0.30
		3	70	32	45.7	2.2	1.2	3.9	0.01	2.1	1.2	3.7	0.014
		4–6^b^	90	23	25.6	1.2	0.6	2.3	0.56	1.2	0.6	2.2	0.60
		7	52	11	21.2	Reference				Reference			
FBSS		No	303	99	32.7	Reference							
		Yes	31	13	41.9	1.3	0.8	2.0	0.27				
Spondylolisthesis		No	205	73	35.6	Reference							
		Yes	129	39	30.2	0.8	0.6	1.2	0.32				
Degenerative lumbar scoliosis		No	270	96	35.6	Reference							
		Yes	64	16	25.0	0.7	0.4	1.1	0.13				
Diabetes mellitus		No	243	83	34.2	Reference							
		Yes	91	29	31.9	0.9	0.7	1.3	0.70				
ASA physical status		1	36	11	30.6	Reference							
		2	257	89	34.6	1.1	0.7	1.9	0.64				
		3	41	12	29.3	1.0	0.5	1.9	0.90				
Surgical duration (min)		<120	157	52	33.1	Reference							
		≥120	177	60	33.9	1.0	0.8	1.4	0.88				
Intraoperative blood loss volume (mL)		<100	145	45	31.0	Reference							
		≥100	189	67	35.5	1.3	0.9	1.8	0.21				
Surgical procedure		Without fusion	181	62	34.3	Reference							
		With fusion	153	50	32.7	1.1	0.8	1.6	0.40				
Radiographic parameter	SVA (mm)	<50	179	60	33.5	Reference							
		≥50	155	52	33.6	1.0	0.7	1.4	0.99				
	TK (°)	≤40	257	87	33.9	Reference							
		>40	77	25	32.5	1.0	0.7	1.4	0.82				
	PI–LL (°)	≤10	128	45	35.2	Reference							
		>10	206	67	32.5	0.9	0.7	1.3	0.62				

^a^ We applied a Poisson regression model and adjusted for age, sex, and statistically significant factors in the univariable model. ^b^ The incidence was similar among Groups 4, 5, and 6, and the sample size for each group was small; therefore, we combined the three groups in this analysis. BMI, body mass index; FBSS, failed back surgery syndrome; ASA, American Society of Anesthesiologists; SVA, sagittal vertical axis; TK, thoracic kyphosis; PI, pelvic incidence; LL, lumbar lordosis; PI–LL, difference between PI and LL (used to evaluate sagittal alignment)

**Table 6:** Poisson regression model of poor surgical improvement

			Patients (n)	Incidents (n)	Incidence (%)	Univariable model				Multivariable model^a^			
						Relative risk	95% Confidence interval		p-value	Relative risk	95% Confidence interval		p-value
Age (years)		65–69	85	15	25.9	Reference				Reference			
		70–79	183	62	33.9	1.3	0.8	2.1	0.27	1.3	0.8	2.0	0.35
		≥80	93	35	37.6	1.5	0.9	2.4	0.15	1.3	0.8	2.1	0.34
Sex		Male	173	53	30.6	Reference				Reference			
		Female	161	59	36.7	1.2	0.9	1.6	0.25	1.2	0.9	1.6	0.30
BMI (kg/m^2^)		<25	228	67	29.4	Reference				Reference			
		≥25	106	45	42.5	1.4	1.1	1.9	0.02	1.4	1.0	1.8	0.049
Distribution patterns of the three preoperative visual analogue scale scores		1	59	27	45.8	2.2	1.2	3.9	0.01	1.9	1.0	3.5	0.047
		2	63	19	30.2	1.4	0.7	2.7	0.28	1.3	0.7	2.5	0.43
		3	70	32	45.7	2.2	1.2	3.9	0.01	1.9	1.1	3.5	0.030
		4–6^b^	90	23	25.6	1.2	0.6	2.3	0.56	1.1	0.6	2.0	0.83
		7	52	11	21.2	Reference				Reference			
FBSS		No	303	99	32.7	Reference							
		Yes	31	13	41.9	1.3	0.8	2.0	0.27				
Spondylolisthesis		No	205	73	35.6	Reference							
		Yes	129	39	30.2	0.8	0.6	1.2	0.32				
Degenerative lumbar scoliosis		No	270	96	35.6	Reference							
		Yes	64	16	25.0	0.7	0.4	1.1	0.13				
Diabetes mellitus		No	243	83	34.2	Reference							
		Yes	91	29	31.9	0.9	0.7	1.3	0.70				
ASA physical status		1	36	11	30.6	Reference							
		2	257	89	34.6	1.1	0.7	1.9	0.64				
		3	41	12	29.3	1.0	0.5	1.9	0.90				
Surgical duration (min)		<120	157	52	33.1	Reference							
		≥120	177	60	33.9	1.0	0.8	1.4	0.88				
Intraoperative blood loss volume (mL)		<100	145	45	31.0	Reference							
		≥100	189	67	35.5	1.3	0.9	1.8	0.21				
Surgical procedure		Without fusion	181	62	34.3	Reference							
		With fusion	153	50	32.7	1.1	0.8	1.6	0.40				
Operated levels		1	107	27	25.2	Reference				Reference			
		2	113	41	36.3	1.4	0.96	2.2	0.08	1.3	0.9	2.0	0.16
		≥3	114	44	38.6	1.5	1.0	2.3	0.04	1.5	0.99	2.2	0.06
Radiographic parameter	SVA (mm)	<50	179	60	33.5	Reference							
		≥50	155	52	33.6	1.0	0.7	1.4	0.99				
	TK (°)	≤40	257	87	33.9	Reference							
		>40	77	25	32.5	1.0	0.7	1.4	0.82				
	PI–LL (°)	≤10	128	45	35.2	Reference							
		>10	206	67	32.5	0.9	0.7	1.3	0.62				

^a^ We applied a Poisson regression model and adjusted for age, sex, and statistically significant factors in the univariable model. BMI, body mass index; FBSS, failed back surgery syndrome; ASA, American Society of Anesthesiologists; SVA, sagittal vertical axis; TK, thoracic kyphosis; PI, pelvic incidence; LL, lumbar lordosis; PI–LL, difference between PI and LL (used to evaluate sagittal alignment)
