# Supplementary material for: Can preoperative visual analogue scale chart patterns predict surgical outcomes in older adults with lumbar spinal stenosis? : A two-center retrospective study
Source: Fujita Med J. 2025 Nov 5;12(1):12–9. doi: 10.20407/fmj.2025-015 (PMC12865282; doi:10.20407/fmj.2025-015)
Supplement: Supplementary file 1 — Supplementary Table [file fmj-12-012-s001.pdf]

Supplementary Table 1. Comparison of baseline characteristics between two groups (n = 181)

		Control (n = 119)	Poor surgical improvement (n = 62)	p value
Age (years)		75.9 ± 5.8	77.2 ± 5.8	0.16
Sex	Male	75	34	0.29
	Female	44	28	
BMI (kg/m^2)		23.7 ± 3.7	24.4 ± 3.8	0.82
Distribution patterns of the three preoperative visual analogue scale scores	1	20 (16.8%)	14 (22.6%)	0.23
	2	24 (20.2%)	12 (19.4%)	
	3	16 (13.4%)	14 (22.6%)	
	4–6[a]	38 (31.9%)	17 (27.4%)	
	7	21 (17.6%)	5 (8.1%)	
FBSS		3 (2.5%)	4 (6.5%)	0.19
Spondylolisthesis		20 (16.8%)	11 (17.7%)	0.87
Degenerative lumbar scoliosis		19 (16.0%)	7 (11.2%)	0.4
Diabetes mellitus		34 (28.6%)	17 (27.4%)	0.87
ASA physical status	1	11	4	0.37
	2	89	52	
	3	19	6	
Surgical duration (min)		88.8 ± 37.6	91.7 ± 37.7	0.90
Intraoperative blood loss volume (mL)		105.2 ± 140.9	92.2 ± 92.9	0.30
Operated levels	1	38	14	0.40
	2	36	20	
	≥3	45	28	
Radiographic parameter	SVA (mm)	53.5 ± 47.6	63.2 ± 54.2	0.21
	TK (°)	32.2 ± 11.2	32.5 ± 12.6	0.23
	PI−LL (°)	14.6 ± 12.8	14.5 ± 15.5	0.24

Data are presented as n, n (%), or mean ± standard deviation.

BMI, body mass index; FBSS, failed back surgery syndrome; ASA, American Society of Anesthesiologists; SVA, sagittal vertical axis; TK, thoracic kyphosis; PI, pelvic incidence; LL, lumbar lordosis; PI−LL, difference between PI and LL (used to evaluate sagittal alignment)

[a]The incidence was similar among Groups 4, 5, and 6, and the sample size for each group was small; therefore, we combined the three groups in this analysis.

t-test or chi-square test
